# QMAC-DST for Rapid Detection of Drug Resistance in Pulmonary Tuberculosis Patients: A Multicenter Pre–Post Comparative Study

**DOI:** 10.3390/jcm13102941

**Published:** 2024-05-16

**Authors:** Nakwon Kwak, Sangyeop Lee, Suyeoun Kim, Eunbee Song, Jae-Joon Yim, Tae Sun Shim, Doosoo Jeon, Byung Woo Jhun, Kwang-Hyuk Seok, Saerom Kim, Sunghoon Kwon, Jeongha Mok

**Affiliations:** 1 Division of Pulmonary and Critical Care Medicine, Department of Internal Medicine, Seoul National University Hospital, Seoul 03080, Republic of Korea; n.kwak@snu.ac.kr (N.K.);; 2 Department of Internal Medicine, Seoul National University College of Medicine, Seoul 03080, Republic of Korea; 3 QuantaMatrix Inc., 131 Gasan digital 1-ro, Geumcheon-gu, Seoul 08506, Republic of Korea; sylee@quantamatrix.com (S.L.);; 4 Department of Pulmonary and Critical Care Medicine, Asan Medical Center, University of Ulsan College of Medicine, Seoul 05505, Republic of Korea; 5 Department of Internal Medicine, Pusan National University Yangsan Hospital, Yangsan 50612, Republic of Korea; 6 Department of Internal Medicine, Pusan National University School of Medicine, Busan 49241, Republic of Korea; 7 Division of Pulmonary and Critical Care Medicine, Department of Medicine, Samsung Medical Center, Sungkyunkwan University School of Medicine, Seoul 06351, Republic of Korea; 8 Department of Laboratory Medicine, The Korean Institute of Tuberculosis, Cheongju 28158, Republic of Korea; 9 Department of Internal Medicine, Pusan National University Hospital, 179 Gudeok-ro, Seo-gu, Busan 49241, Republic of Korea; 10 Biomedical Research Institute, Pusan National University Hospital, Busan 49241, Republic of Korea

**Keywords:** drug susceptibility test, QMAC-DST, resistance, tuberculosis

## Abstract

**Background/Objectives:** This study explores the impact of QMAC-DST, a rapid, fully automated phenotypic drug susceptibility test (pDST), on the treatment of tuberculosis (TB) patients. **Methods:** This pre–post comparative study, respectively, included pulmonary TB patients who began TB treatment between 1 December 2020 and 31 October 2021 (pre-period; pDST using the Löwenstein–Jensen (LJ) DST (M-kit DST)) and between 1 November 2021 and 30 September 2022 (post-period; pDST using the QMAC-DST) in five university-affiliated tertiary care hospitals in South Korea. We compared the turnaround times (TATs) of pDSTs and the time to appropriate treatment for patients whose anti-TB drugs were changed based on these tests between the groups. All patients were permitted to use molecular DSTs (mDSTs). **Results:** A total of 182 patients (135 in the M-kit DST group and 47 in the QMAC-DST group) were included. The median TAT was 36 days for M-kit DST (interquartile range (IQR), 30–39) and 12 days for QMAC-DST (IQR, 9–15), with the latter being significantly shorter (*p* < 0.001). Of the total patients, 10 (5.5%) changed their anti-TB drugs based on the mDST or pDST results after initiating TB treatment (8 in the M-kit DST group and 2 in the QMAC-DST group). In the M-kit DST group, three (37.5%) patients changed anti-TB drugs based on the pDST results. In the QMAC-DST group, all changes were due to mDST results; therefore, calculating the time to appropriate treatment for patients whose anti-TB drugs were changed based on pDST results was not feasible. In the QMAC-DST group, 46.8% of patients underwent the first-line line probe assay compared to 100.0% in the M-kit DST group (*p* < 0.001), indicating that rapid QMAC-DST results provide quicker assurance of the ongoing treatment by confirming susceptibility to the current anti-TB drugs. **Conclusions:** QMAC-DST delivers pDST results more rapidly than LJ-DST, ensuring faster confirmation for the current treatment regimen.

## 1. Introduction

Tuberculosis (TB) continues to be one of the most significant infectious diseases, representing a serious threat to global public health. In 2022, ~7.5 million new TB cases were reported worldwide, marking the highest number since the World Health Organization (WHO) began its global TB monitoring in 1995 [1]. In the same year, TB was responsible for ~1.3 million deaths globally [1]. These high incidence and mortality rates markedly diverge from the targets set by the WHO’s End TB Strategy, which aims to achieve a 50% reduction in TB incidence rates and 75% reduction in TB-related deaths by 2025, relative to 2015 levels. The increasing prevalence of drug-resistant TB further complicates efforts to eradicate the disease.

The rapid and accurate diagnosis of drug-resistant TB is critical for improving patient outcomes, as it allows for the prompt initiation of suitable treatment regimens. It also plays a vital role in minimizing the development of acquired drug resistance and the transmission of drug-resistant *Mycobacterium tuberculosis* (MTB) within communities [2,3]. Traditionally, the culture-based phenotypic drug susceptibility test (pDST) has been the standard method for detecting drug-resistant TB. However, the effectiveness of pDST is limited by the slow growth rate of MTB, which leads to delays in the confirmation of resistance. Results from DST using the Löwenstein–Jensen (LJ) medium are typically available within 4–6 weeks. By contrast, the Mycobacteria Growth Indicator Tube (MGIT)-DST, another culture-based pDST, provides results in 1–2 weeks, which is significantly faster than LJ-DST. Nevertheless, MGIT-DST is limited to first-line anti-TB drugs [4,5,6,7].

Molecular DST (mDST) provides the advantage of quickly identifying genetic mutations associated with resistance in MTB, thus enabling prompt diagnosis and the initiation of treatment for patients with drug-resistant TB. However, mDST, much like MGIT-DST, is limited to a narrow range of drugs and mainly detects common genetic mutations, resulting in suboptimal sensitivity [8]. Whole-genome sequencing (WGS) theoretically has the potential to overcome these limitations. However, its implementation is impeded by the requirement for expensive equipment and highly trained personnel, making its widespread use in clinical practice a considerable challenge. Furthermore, the bioinformatics needed for analyzing drug resistance in WGS data are not yet sufficiently developed, which adds to the existing barriers [7,9,10,11]. In conclusion, there is an urgent need for a novel DST method that can rapidly determine resistance to a wider range of anti-TB drugs to adequately address the issue of drug-resistant TB.

The recently developed QMAC-DST (QuantaMatrix, Seoul, Republic of Korea) is a rapid, fully automated pDST. By using a microfluidic chip and imaging technologies, QMAC-DST is capable of determining resistance to >15 anti-TB drugs within a week using a culture specimen [12]. Its high concordance with the conventional LJ-DST has been validated in previous studies [13,14,15]. This multicenter study seeks to investigate the impact of QMAC-DST on the treatment of pulmonary TB patients, with a particular focus on comparing QMAC-DST and LJ-DST.

## 2. Materials and Methods

### 2.1. Study Design and Population

This pre–post comparative study was conducted in five university-affiliated tertiary care hospitals in South Korea. The participating hospitals are well equipped for diagnosing and treating TB, with TB specialists on staff, advanced laboratories, and negative-pressure rooms. These hospitals serve as referral hospitals for all provincial patients with TB. This study included patients aged 19–85 years who began pulmonary TB treatment between 1 December 2020 and 31 October 2021 (pre-period; pDST using the LJ-DST [M-kit DST]) and between 1 November 2021 and 30 September 2022 (post-period; pDST using the QMAC-DST). Patients were excluded if they had only extrapulmonary TB, requested pDST before enrollment, started TB treatment >2 weeks before enrollment, were unable to undergo pDST due to non-cultivation of MTB within 12 weeks of starting treatment, had invalid pDST results, or did not adhere to clinical visits and sputum tests at 4-week intervals for up to 12 weeks after starting treatment. The inclusion and exclusion criteria were consistent for both periods.

The study protocol was approved by the Institutional Review Board of each participating hospital. In the pre-period, the requirement for informed consent was waived owing to the retrospective nature of the study; in the post-period, written informed consent was obtained from all participants. This study is registered with the CRIS registry (KCT0006891).

### 2.2. Data Collection

Patient data were collected retrospectively for the pre-period and prospectively for the post-period, including demographics (age, sex, height, weight), medical history (previous TB treatments, comorbidities), results of chest radiographs and computed tomography scans, information regarding mDST and pDST (request dates, notification dates of results, and DST results), information on TB treatment (date of treatment commencement, initial regimen, sputum test results at each visit), and changes in anti-TB drug based on DST results along with the dates of the changes.

### 2.3. Drug Susceptibility Test

Each participating hospital was allowed to continue using the mDST methods already in use (e.g., Xpert MTB/RIF assay, first-line line probe assay (LPA), or fluoroquinolone target gene sequencing) or MGIT-DST. The various mDSTs and MGIT-DST were conducted according to the manufacturer’s instructions. pDST (M-kit DST or QMAC-DST) was requested immediately upon confirmation of a positive culture result, either at the start of treatment or during the course of treatment.

In the pre-period, pDST was conducted using the absolute concentration method with LJ medium (M-kit DST; The Korean Institute of Tuberculosis, Cheongju, Republic of Korea). The drugs and their critical concentrations for resistance determination were as follows: isoniazid (INH), 0.2 and 1.0 μg/mL; rifampin (RIF), 40 μg/mL; ethambutol (EMB), 2.0 μg/mL; rifabutin (RFB), 20 μg/mL; ofloxacin (OFX), 4.0 μg/mL; levofloxacin (LFX), 2.0 μg/mL; moxifloxacin (MFX), 1.0 μg/mL; streptomycin (SM), 10 μg/mL; amikacin (AMK), 30 μg/mL; kanamycin (KM), 30 μg/mL; capreomycin (CM), 40 μg/mL; prothionamide (PTO), 40 μg/mL; cycloserine (CS), 30 μg/mL; p-aminosalicylic acid (PAS), 1.0 μg/mL; and linezolid (LZD), 2.0 μg/mL.

In the post-period, pDST was conducted using QMAC-DST. The process included the reference strain H37Rv (ATCC 27294) for quality control. Cells derived from LJ medium colonies underwent thorough testing. Bacterial growth images were systematically captured using a 10× microscope lens over a period of 0–7 days through a time-lapse methodology. Following image acquisition, an algorithmic approach was used for subsequent processing and analysis. Further details regarding experimental methodologies and procedures are available in a previous publication [15]. The critical concentrations for drug resistance determination were set as follows: INH, 0.1 μg/mL; RIF, 1.5 μg/mL; EMB, 5 μg/mL; RFB, 1.25 μg/mL; OFX, 2 μg/mL; LFX, 0.75 μg/mL; MFX, 0.5 μg/mL; SM, 1.0 μg/mL; AMK, 2 μg/mL; KM, 2.5 μg/mL; CM, 2.5 μg/mL; PTO, 2.5 μg/mL; CS, 16 μg/mL; PAS, 4 μg/mL; and LZD, 1.0 μg/mL. Pyrazinamide (PZA) susceptibility was determined using the pyrazinamidase test in both periods.

### 2.4. Treatment and Follow-Up

Patients initiated TB treatment based on acid-fast bacilli (AFB) smear, MTB culture, TB-polymerase chain reaction (PCR) results (either conventional TB-PCR or Xpert MTB/RIF assay), or clinical decision making based on clinical and radiological findings. The initial treatment regimen was in accordance with the Korean TB guidelines, which align with WHO guidelines. For instance, if the Xpert MTB/RIF assay showed no RIF resistance, the treatment involved a four-drug regimen, including INH, RIF, EMB, and PZA. In cases of RIF resistance, the regimen consisted of a fluoroquinolone, bedaquiline (BDQ), LZD, and group B drugs. Following treatment initiation, if resistance to any drugs in the regimen was detected in subsequent mDST or pDST, resistant drugs were either substituted with different drugs or discontinued as soon as possible, based on the earlier test results, regardless of the DST type. Patients visited the hospital at 4-week intervals for sputum AFB smear and culture tests up to 12 weeks after initiating treatment (the 12-week period was set for research purposes, while patients received standard care according to the guideline-specified duration).

### 2.5. Outcome

In this study, the primary outcome was the time to appropriate treatment for patients who underwent changes in their anti-TB drug regimen based on the pDST (M-kit DST or QMAC-DST) results. Time to appropriate treatment was defined as the interval (in days) from the initiation of TB treatment to the last modification of anti-TB drugs, occurring within the first 12 weeks after starting treatment. Secondary outcomes included the turnaround time (TAT) for each pDST (time from test request to notification of test results (in days)), the proportion of patients who had their anti-TB drug regimen changed based on mDST or pDST results, and the time to sputum smear and culture conversion for these patients.

### 2.6. Statistical Analysis

Continuous variables are presented as medians with interquartile ranges (IQRs), and categorical variables as numbers with percentages. Continuous variables were compared using the independent *t*-test or the Mann–Whitney U test, and categorical variables using the chi-squared test or Fisher’s exact test, as appropriate. In all analyses, *p* < 0.05 was considered indicative of statistical significance. All statistical analyses were performed using SPSS Statistics version 25.0 (SPSS, Chicago, IL, USA).

## 3. Results

### 3.1. Characteristics of Study Participants

After applying the predefined inclusion and exclusion criteria, a total of 182 patients were included in the final analysis: 135 in the M-kit DST group and 47 in the QMAC-DST group. The median age of these patients was 61.0 years, and 60.4% were male. Hypertension was the most common comorbidity, affecting 23.6% (n = 43) of the patients. Previous TB treatment history was noted in 10.4% (n = 19) of the total patients. The sputum AFB smear positivity rate was 31.9%. When comparing the baseline characteristics of the two groups, it was observed that patients in the M-kit DST group were older and had a higher proportion of malignancy as a comorbidity (Table 1).

### 3.2. Testing Rates, TAT, and Results of DSTs

Table 2 presents the DSTs conducted in both groups and the proportion of patients who underwent each DST. Of the total number of patients, 165 (90.7%) underwent the Xpert MTB/RIF assay, and 157 (86.3%) underwent the first-line LPA. A lower proportion of patients in the QMAC-DST group underwent the first-line LPA compared to the M-kit DST group (46.8% vs. 100.0%, *p* < 0.001). The median TAT for M-kit DST was 36.0 days (IQR, 30.0–39.0), whereas it was 12.0 days (IQR, 9.0–15.0) for QMAC-DST; thus, the TAT for QMAC-DST was significantly shorter (*p* < 0.001) (Table 2).

Table 3 shows the results of M-kit DST and QMAC-DST for both groups. The most commonly identified resistant drug was INH (11.5%, n = 21), followed by RIF (7.1%, n = 13). There was no significant difference in the frequency of resistant drugs between the two groups.

### 3.3. Anti-TB Drug Change Based on DST Results

Of all the patients, 10 (5.5%) changed or discontinued their anti-TB drugs based on the DST results after initiating TB treatment (8 patients in the M-kit DST group and 2 in the QMAC-DST group). In the M-kit DST group, changes or discontinuations were due to the following: INH resistance on first-line LPA (n = 4); INH resistance on M-kit DST (n = 1); EMB resistance on M-kit DST (n = 1); LFX resistance on M-kit DST (n = 1); RIF resistance on Xpert MTB/RIF assay (n = 1); and PZA resistance on the pyrazinamidase test (n = 1). In the QMAC-DST group, two patients changed their anti-TB drugs following the identification of INH resistance on first-line LPA (Table 4). However, no changes in anti-TB drugs based on the QMAC-DST results were recorded. Therefore, a comparison of the time to appropriate treatment for patients who changed their anti-TB drugs based on pDST results between the two groups was not possible.

## 4. Discussion

In this study, the median TAT for QMAC-DST was 12 days, significantly faster compared to the 36 days required for M-kit DST. However, the inability to compare the time to appropriate treatment for patients who changed their anti-TB drugs based on pDST results between the groups was a limitation, as no patients changed their anti-TB drug regimen based on the QMAC-DST results. This was the primary outcome of this study. Unfortunately, the enrollment of TB patients during the post-period was limited due to the COVID-19 pandemic, resulting in a reduced number of patients with a variety of resistance patterns in the QMAC-DST group. Despite this, the substantial difference in TATs between M-kit DST and QMAC-DST suggests that a more significant difference in the primary outcome might have been observed with a larger patient cohort during the post-period. Additionally, the rapid determination of susceptibility to the current anti-TB drugs through QMAC-DST provided quicker confirmation of the ongoing treatment regimen. For example, in our study, the frequency of conducting first-line LPA was lower in the QMAC-DST group. This likely occurred due to the rapid availability of QMAC-DST results, particularly in patients with smear-negative/culture-positive TB, reducing the need for additional first-line LPA using culture specimens.

In our study, among the eight patients in the M-kit DST group who changed or discontinued their anti-TB drugs, three (37.5%) did so based on the pDST (M-kit DST) results. This finding demonstrates that, despite the introduction of various mDST methods for rapid resistance identification and treatment modification, a significant number of patients still rely on pDST results. The currently available mDST methods are limited in their range of targeted drugs and ability to detect uncommon mutations. Furthermore, when mDST and pDST results are inconsistent, interpreting genotype–phenotype relationships becomes complex due to gaps in our understanding of the resistance genetics database. Additionally, certain types of resistance, such as those arising from efflux pump activation, reduced cell wall permeability, or heteroresistance, are difficult to detect using mDST [16,17,18]. Therefore, mDST cannot yet fully replace pDST, highlighting the continuing importance of pDST.

To overcome the lengthy TAT of the current LJ-DST, various commercial and non-commercial pDST methods are in use. The MGIT-DST offers a quicker TAT of 1–2 weeks; however, it is commercially available only for testing first-line anti-TB drugs, and its results are influenced by factors, such as culture purity, inoculum size, and contamination. Additionally, MGIT-DST presents challenges due to its technical complexity, need for laboratory infrastructure, and personnel training requirements [19,20,21]. Other non-commercial culture-based pDST methods include the microscopic observation drug susceptibility assay, nitrate reductase assay, and colorimetric redox indicator methods. These tests also facilitate the rapid detection of drug resistance but are typically limited to first-line anti-TB drugs, are labor-intensive, and may pose safety concerns. They also depend on the skill of laboratory personnel for result interpretation and are not efficient for processing large sample volumes [22,23,24,25]. QMAC-DST addresses several limitations of traditional pDST by offering rapid TAT, the ability to test various first- and second-line drugs, high throughput, reduced labor intensity, effectiveness regardless of inoculum size, enhanced safety with sealing film and a locking lid, along with an agarose matrix embedding the MTB and elimination of errors due to the MTB tracking failure [12,13,14,15]. However, because QMAC-DST uses colonies derived from the LJ medium, the timing for the confirmation of QMAC-DST results after the start of treatment can be delayed, depending on the time required for the MTB culture. A recent study showed that when QMAC-DST is coupled with a liquid medium, the test results can be confirmed more quickly [15].

The recent reclassification of core drugs in longer regimens for MDR-TB treatment has led to a revision in the definitions of pre-extensively drug-resistant TB and extensively drug-resistant TB [26]. Additionally, the introduction of shorter treatment regimens (e.g., “BPaL regimen”, “BPaLM regimen”, and “MDR-END regimen”) has highlighted the need for rapid and accurate diagnosis of resistance to new anti-TB drugs, such as BDQ, delamanid (DLM), and pretomanid (PA), as well as repurposed drugs including LZD [27,28]. Currently, however, there are no validated mDSTs for BDQ, DLM, or LZD, with reliance on traditional, time-consuming pDST methods. Additionally, no commercially available DST is available for PA. QMAC-DST shows promise in rapidly detecting resistance to LZD, and with further validation, it could potentially be used for BDQ, DLM, and PA as well.

This study has several limitations. First, as previously mentioned, the post-period included fewer patients, preventing a comprehensive evaluation and comparison of the time to appropriate treatment. However, the rapid TAT of QMAC-DST demonstrated in this study may potentially facilitate quicker regimen adjustments for patients with drug-resistant TB. Second, there is an inherent limitation in our study’s pre–post comparison design. However, except for the pDST used in both groups (M-kit DST vs. QMAC-DST), the entire diagnostic and treatment process for patients was identical in both groups. Third, our study permitted the use of all mDSTs, which may have limited the ability to compare the effectiveness of the different pDSTs (M-kit DST vs. QMAC-DST). However, this approach was chosen to mirror real-world practice, where various mDSTs are widely used. Fourth, in the hospitals where the study was conducted, the second-line LPA was not widely available, which could have influenced the study results. Lastly, the TAT for QMAC-DST in our study was longer than the 1-week duration reported in previous studies. This discrepancy was due to administrative delays in specimen reception and reporting, highlighting the need for improvements through stewardship.

In conclusion, QMAC-DST provided faster pDST results compared to M-kit DST, and the rapid determination of susceptibility to the currently used anti-TB drugs through QMAC-DST can offer quicker assurance regarding the ongoing treatment regimen. Further research is needed to evaluate the impact of rapid resistance confirmation using QMAC-DST on the ability to swiftly modify treatment regimens, and treatment outcomes, in patients with drug-resistant TB.

## Figures and Tables

**Table 1:** Baseline characteristics of the study participants.

	M-Kit DST Group (n = 135)	QMAC-DST Group (n = 47)	Total (N = 182)	*p*-Value *
Age, y	63.0 [55.0–73.0]	53.0 [47.0–64.0]	61.0 [50.8–70.3]	0.001
Sex, male	79 (58.5)	31 (66.0)	110 (60.4)	0.369
Comorbidity				
Hypertension	31 (23.0)	12 (25.5)	43 (23.6)	0.721
Diabetes mellitus	32 (23.7)	8 (17.0)	40 (22.0)	0.341
Malignancy	26 (19.3)	1 (2.1)	27 (14.8)	0.004
HIV	0 (0.0)	0 (0.0)	0 (0.0)	N/A
History of previous TB treatment	15 (11.1)	4 (8.5)	19 (10.4)	0.785
Sputum AFB smear, positive	42 (31.1)	16 (34.0)	58 (31.9)	0.710
Radiologic finding				
Cavity	42 (31.1)	15 (31.9)	57 (31.3)	0.918
Bilateral lungs involvement	45 (33.3)	10 (21.3)	55 (30.2)	0.121

Data are presented as median (IQR) for continuous variables and number (%) for categorical variables. * Comparison between M-kit DST and QMAC-DST groups. AFB, acid-fast bacilli; HIV, human immunodeficiency virus; TB, tuberculosis. N/A: Not applicable.

**Table 2:** Testing rates and turnaround times of drug susceptibility tests in all participants.

	M-Kit DST Group (n = 135)	QMAC-DST Group (n = 47)	Total (N = 182)	*p*-Value *
Drug susceptibility test, performed				
Xpert MTB/RIF assay	120 (88.9)	45 (95.7)	165 (90.7)	0.245
First-line line probe assay	135 (100.0)	22 (46.8)	157 (86.3)	<0.001
Fluoroquinolone target gene sequencing	9 (6.7)	5 (10.6)	14 (7.7)	0.358
MGIT-DST	0 (0.0)	9 (19.1)	9 (4.9)	<0.001
M-kit DST	135 (100.0)	0 (0.0)	135 (74.2)	N/A
QMAC-DST	0 (0.0)	47 (100.0)	47 (25.8)	N/A
Turnaround time, days ^†^				
Xpert MTB/RIF assay	0.0 [0.0–1.0]			
First-line line probe assay	9.0 [5.0–29.5]			
Fluoroquinolone target gene sequencing	14.0 [13.0–18.3]			
MGIT-DST	30.0 [21.5–43.5]			
M-kit DST ^‡^	36.0 [30.0–39.0]			
QMAC-DST ^‡^	12.0 [9.0–15.0]			

Data are presented as median (IQR) for continuous variables and number (%) for categorical variables. * Comparison between M-kit DST and QMAC-DST groups. ^†^ Time from test request to notification of test results. ^‡^ Comparison between M-kit DST and QMAC-DST (*p* < 0.001). DST, drug susceptibility test; MGIT, mycobacteria growth indicator tube. N/A: Not applicable.

**Table 3:** Results of M-kit DST and QMAC-DST.

Resistant Drug	M-Kit DST Group (n = 135)	QMAC-DST Group (n = 47)	Total (N = 182)	*p*-Value *
Isoniazid	15 (11.1)	6 (12.8)	21 (11.5)	0.760
Rifampin	11 (8.1)	2 (4.3)	13 (7.1)	0.520
Ethambutol	7 (5.2)	2 (4.3)	9 (4.9)	>0.999
Pyrazinamide ^†^	8 (5.9)	0 (0.0)	8 (4.4)	0.115
High dose isoniazid	5 (3.7)	4 (8.5)	9 (4.9)	0.240
Rifabutin	7 (5.2)	2 (4.3)	9 (4.9)	>0.999
Ofloxacin	1 (0.7)	0 (0.0)	1 (0.5)	>0.999
Levofloxacin	1 (0.7)	0 (0.0)	1 (0.5)	>0.999
Moxifloxacin	1 (0.7)	0 (0.0)	1 (0.5)	>0.999
Streptomycin	5 (3.7)	2 (4.3)	7 (3.8)	>0.999
Amikacin	0 (0.0)	0 (0.0)	0 (0.0)	N/A
Kanamycin	0 (0.0)	0 (0.0)	0 (0.0)	N/A
Capreomycin	0 (0.0)	0 (0.0)	0 (0.0)	N/A
Prothionamide	2 (1.5)	1 (2.1)	3 (1.6)	>0.999
Cycloserine	0 (0.0)	1 (2.1)	1 (0.5)	0.258
Para-aminosalicylic acid	0 (0.0)	1 (2.1)	1 (0.5)	0.258
Linezolid	0 (0.0)	0 (0.0)	0 (0.0)	N/A

Data are presented as number (%). * Comparison between M-kit DST and QMAC-DST groups. ^†^ Using the pyrazinamidase test. N/A: Not applicable.

**Table 4:** Anti-TB drug change based on the drug susceptibility test results.

	Group	Initial Regimen	Resistant Drug (Drug Susceptibility Test)	Time from Treatment Initiation to Drug Change, Day	Discontinued Drug	Added Drug
Patient 1	QMAC	H, R, E, Z	H (FL-LPA)	37	H	Lfx
Patient 2	QMAC	H, R, E, Z	H (FL-LPA)	21	H	Lfx
Patient 3 *	M-kit	H, R, E, Z	H (FL-LPA)	26	H	Lfx
	M-kit	H, R, E, Z	Z (pyrazinamidase test)	89	Z	none
Patient 4	M-kit	R, E, Z, Lfx	Lfx (M-kit)	71	Lfx	none
Patient 5	M-kit	H, R, E, Z	H (FL-LPA)	27	H	Lfx
Patient 6	M-kit	H, R, E, Z	H (FL-LPA)	11	H	Lfx
Patient 7	M-kit	H, R, E, Z	H (M-kit)	56	H	Lfx
Patient 8	M-kit	H, R, E, Z	E (M-kit)	64	E	Lfx
Patient 9	M-kit	H, R, E, Z	R (Xpert MTB/RIF)	6	H, R, E	Lfx, Amk
Patient 10	M-kit	H, R, E, Z	H (FL-LPA)	11	H	Lfx

* Patient 3 changed anti-TB drugs twice during the treatment period. Amk, amikacin; E, ethambutol; FL-LPA, first-line line probe assay; H, isoniazid; Lfx, levofloxacin; R, rifampin; Z, pyrazinamide.

## Data Availability

All relevant data are within the paper.
